# Research on accuracy enhancement methods for highway slope deformation monitoring based on the BeiDou system

**DOI:** 10.1371/journal.pone.0328158

**Published:** 2025-07-14

**Authors:** Yuhong Luo, Geyu Chen

**Affiliations:** 1 Dr., School of Civil Engineering, Chongqing Jiaotong University, Chongqing, China; 2 Engineer, China Merchants Chongqing Highway Engineering Testing Center Co., Ltd., Chongqing, China; Beijing Institute of Technology, CHINA

## Abstract

To address the issue of low accuracy caused by multipath effects in BeiDou satellite-based slope monitoring, this study proposes a multipath error mitigation technique based on the BeiDou Navigation Satellite System (BDS). By introducing a real-time error correction mechanism, the proposed method significantly reduces the impact of multipath effects on positioning accuracy and deformation monitoring precision. The research methodology comprises multipath effect analysis and modeling, a real-time error correction mechanism based on correction signals, and further enhancement of accuracy and stability through the integration of Kalman filtering and multi-source data fusion optimization techniques. Experiments conducted under various environmental conditions validate the effectiveness of the proposed multipath error mitigation approach. The results demonstrate that in complex terrains such as mountainous areas and canyons, the positioning accuracy improved by 53.53% to 66.40% after applying correction signals, while the deformation monitoring accuracy increased by 50.62% to 60.10%. Furthermore, through multi-source data fusion optimization, the improved positioning accuracy reached a 71.16% enhancement, and deformation monitoring accuracy was improved by 66.84%. The experimental findings strongly confirm the significant advantages of the real-time error correction technique based on the BeiDou system in improving the accuracy of deformation monitoring.

## 1. Introduction

The timeliness and accuracy of road slope deformation monitoring are not only the basis for engineering design and construction but also key to ensuring the safe operation of highways [1–3]. Traditional methods of slope deformation monitoring mainly rely on manual observation and conventional measurement technologies, such as optical measurement and manual leveling. Although these methods can provide deformation data to some extent, they are often limited by low measurement frequency, insufficient monitoring precision, and high consumption of human and material resources. Particularly in complex environments, traditional methods fail to meet the demands of modern highway slope deformation monitoring in terms of accuracy and response speed [4–6]. Against this backdrop, the Global Navigation Satellite System (GNSS), especially the BeiDou Navigation Satellite System (BDS), has gradually become one of the core technologies in slope deformation monitoring due to its high precision, efficiency, all-weather capability, and automation advantages [7,8]. However, the application of the BeiDou system also faces several challenges, one of the most prominent being multipath effects. Multipath effects occur when satellite signals are reflected or refracted by surrounding buildings, mountains, water surfaces, etc., causing the received signal path to be longer than the direct path, resulting in positioning errors [9,10]. This effect is particularly pronounced in mountainous and canyon environments, where satellite signals are significantly affected, severely impacting the positioning accuracy and the reliability of monitoring results from the BeiDou system. Therefore, how to effectively deal with multipath errors in the BeiDou system and improve monitoring precision has become a key technical issue that needs to be addressed in the field of slope deformation monitoring [11,12].

As China’s independently developed global satellite navigation system, the BeiDou system’s precision and reliability have been widely validated in recent years. Firstly, the BeiDou system has significant advantages in real-time, high-precision displacement monitoring. Zhang et al. [13] developed a general monitoring system based on BeiDou and conducted field monitoring of expansive soil slopes. The results indicated that the system maintained good signal stability even in high-humidity environments, making it suitable for slope stability evaluation in disaster-prone areas. Zhou et al. [14] combined BeiDou with time-series InSAR (Interferometric Synthetic Aperture Radar) technology to conduct long-term deformation analysis along the Changgan High-speed Railway, verifying the high sensitivity of BeiDou data in capturing millimeter-level slow changes. Secondly, for high and steep rock slopes, multi-layered slope structures, and complex failure modes influenced by external disturbances, the BeiDou system plays an important role in multi-source fusion monitoring systems. Yang et al. [15] studied the deformation pattern of layered rock slopes induced by stepped excavation, emphasizing the importance of continuous displacement monitoring. Chen et al. [16] explored the deformation patterns of rock slopes in over-excavated fault zones and, by integrating BeiDou data, effectively identified the spatiotemporal evolution of deep sliding surfaces. Leng et al. [17] studied the rainfall-triggered mechanism of loess landslides from field monitoring to indoor simulation systems, highlighting the critical role of high temporal resolution monitoring data in disaster early warning. Under special geological conditions, such as high embankment fills or mining-disturbed areas, slope stability evaluation relies on high-precision three-dimensional displacement monitoring. Zhang et al. [18] used the BeiDou system to monitor the stability changes of high embankment slopes under rainfall conditions, and by combining seepage-stability coupling analysis, they effectively captured potential instability processes. Zhang et al. [19] analyzed the deformation and failure evolution process of rock mass structures at open-pit mine slopes induced by underground mining based on continuous GNSS observation data, highlighting the continuous monitoring advantages of the BeiDou system in such scenarios. With the further improvement of BeiDou system accuracy, its integration with artificial intelligence models is becoming a research hotspot for intelligent slope monitoring. Yang et al. [20] constructed a deep learning model integrating GNSS data, effectively enhancing the accuracy of landslide displacement predictions and providing technical support for intelligent early warning systems. In overseas slope engineering, similar GNSS methods to the BeiDou system have been adopted for slope monitoring. Furlani et al. [21] combined digital photogrammetry with geo-mechanical models to achieve digital recording of coastal cliff slope deformation processes, showing the core role of GNSS in multi-source monitoring platforms. Zhou et al. [22] further proposed a remote real-time monitoring system based on a cloud computing architecture, integrating BeiDou/GNSS data to establish an efficient data transmission and processing mechanism, providing an effective technological path for the remote control of large-scale slope engineering.

The occurrence of multipath effects is a common problem in GNSS positioning systems, especially in complex terrain environments. Various processing techniques have been proposed to address this issue. Common solutions include antenna design and optimization, data processing and multipath elimination algorithms, multi-source fusion technology, precise point positioning (PPP), and post-processing techniques. Antenna design is one of the key methods for addressing multipath effects. By optimizing the antenna structure, the interference from reflected signals can be effectively suppressed, reducing the impact of multipath effects. Optimizing antenna design can fundamentally reduce reflected signal paths during reception, improving the system’s anti-interference capability and positioning accuracy [23]. Regarding multipath error elimination, improvements in data processing methods and algorithms are a current research focus. Zhang et al. [24] proposed a multipath error suppression method based on a hemisphere model, which significantly improved the effect of multipath suppression by modeling and correcting precise positioning data from BDS2/BDS3 (Beidou Navigation Satellite System (2nd Generation)/ Beidou Navigation Satellite System (3rd Generation)). Similarly, Zou et al. [25] proposed a new error suppression method for NLOS (Non-Line-Of-Sight) signals in high-precision GNSS data processing, further improving the accuracy and stability of multipath error correction. In addition to relying solely on GNSS signals, multi-source information fusion technology can also effectively mitigate the impact of multipath effects. Xue et al. [26] reviewed the development of GNSS multipath suppression technologies, particularly methods that fuse GNSS data with other auxiliary sensors such as inertial measurement units (IMU) and optical sensors. These technologies can provide additional positioning information, helping to eliminate the uncertainties caused by multipath errors. Precise point positioning (PPP) technology is another effective method for addressing multipath effects, especially in high-precision applications. Gao et al. [27] proposed a multipath correction method for the Hong Kong–Zhuhai–Macau Bridge monitoring based on PPP-RTK (Precise Point Positioning – Real-Time Kinematic) technology. By combining precise real-time positioning with multipath error correction algorithms, high-precision positioning can be achieved in large-scale engineering monitoring. Furthermore, Lu et al. [28] corrected multipath effects in the BDS3 system using precise point positioning and demonstrated the effectiveness of this method in marine measurements. In urban environments, where high-rise buildings exacerbate multipath effects, Gong et al. [29] proposed a GNSS multipath suppression method based on the *K*-means classification algorithm. By classifying and analyzing reflected signals in urban environments, this method effectively reduces multipath errors. It can significantly improve the positioning accuracy and reliability of GNSS in urban canyons or densely built-up areas. Signal processing techniques, such as filtering methods, are also important tools for suppressing multipath effects. Itoh and Aoki [30] proposed a technique based on sidereal filter in the position domain to reduce multipath errors in 30-second dynamic GPS (Global Positioning System) measurements. This filtering method can dynamically correct positioning errors, especially in high-speed positioning applications.

To address the shortcomings of existing multipath error processing methods, this paper proposes a multipath error processing technology based on the BeiDou system. Specifically, this study integrates real-time positioning from the BeiDou system with high-precision deformation analysis. By introducing a real-time error correction mechanism, it effectively reduces the error impacts caused by multipath effects. Through the introduction of signal correction, this research can provide real-time corrections to BeiDou system positioning errors in complex environments, thus improving the precision of slope deformation monitoring.

## 2. Multipath error processing technology based on the BeiDou system

In complex environments, particularly in areas with significant terrain fluctuations such as mountainous regions and canyons, multipath effects have a substantial impact on the accuracy of the BeiDou system. To address this issue, this paper proposes a multipath error processing technology based on the BeiDou system. This technology combines real-time positioning from the BeiDou system with signal correction to dynamically correct multipath errors, which, in theory, can significantly enhance the precision and reliability of deformation monitoring.

### 2.1. Analysis and modeling of multipath effects

#### 2.1.1. Basic principle of multipath effects.

Multipath effects occur when satellite signals are reflected or refracted by the ground or surrounding obstacles during their propagation, resulting in a longer path for the received signal than for the direct path. This effect not only causes errors in the positioning results but may also affect the precision of deformation monitoring. In complex environments, the reflection and refraction phenomena of signals become more intricate, posing significant challenges to the high-precision positioning of the BeiDou system.

Assuming that a satellite signal is reflected by an obstacle during propagation, multiple paths are formed, including the direct path and the reflected path. The actual propagation distance ρof the signal can be expressed by the following formula:


$$\rho =\rho_{d}+\rho_{r}$$ (1)

Where, $\rho_{d}$ is the propagation distance of the direct path, and $\rho_{r}$ is the propagation distance of the reflected path. Because the propagation time of the reflected path is longer, the signal reaches the receiver later than the direct path, resulting in a positioning error calculated by the receiver. The size of the multipath error is closely related to the distance of the reflected path, the nature of the reflecting object, and the relative position between the satellite and the receiver.

#### 2.1.2. Modeling method of multipath errors.

To accurately correct multipath errors, it is first necessary to establish a mathematical model of the reflected path. Assuming the propagation time of the reflected signal path is $T_{r}$, and the propagation time of the direct path is $T_{d}$, the error in the reflected path ΔT can be expressed as:


$$\Delta T=T_{r}-T_{d}=\frac{\Delta \rho }{c}$$ (2)

Where, c is the speed of light, and Δρ is the distance difference between the reflected path and the direct path. By precisely modeling the propagation time errors of the reflected path, multipath effects can be better corrected.

To handle the multipath effects in complex terrains, this paper introduces a spatial-domain modeling method. This method constructs a multipath error model based on a semi-hemispherical grid network, which effectively reduces the impact of reflected signals and performs real-time error correction based on this model.

#### 2.1.3. The multipath error half-sky grid model.

**Fig 1:**
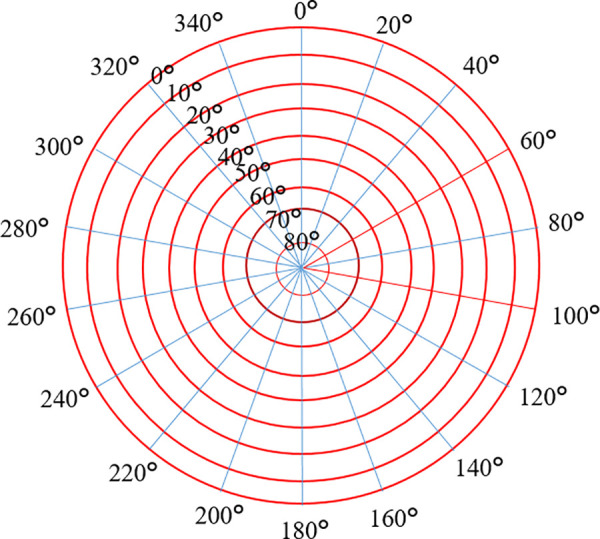
Semi-hemispherical grid model.

The Multipath Error Half-Sky Grid Model (MHGM) is a spatial-domain modeling method primarily used to minimize the impact of multipath effects on positioning accuracy. As shown in Fig 1, the model divides the space into multiple grids, each representing a small area. By modeling and correcting the errors in these areas, positioning accuracy can be significantly improved. The basic idea of the MHGM model is to build an error model based on the satellite’s orbit, the positions of ground obstacles, and the relative position of the receiver, dynamically adjusting the error correction in each grid.

The model can be established through the following steps:

(1) Grid the Ground Area: The space is divided into several small semi-hemispherical grids based on the distribution of ground obstacles. The size of each grid depends on the distribution of obstacles in the ground area.
(2) Analyze Reflected Signals: For each grid, the reflected signals are analyzed, and the time difference between the reflected and direct signal propagation is calculated.
(3) Error Correction: Based on the time difference of the reflected signals, correction terms are introduced into the positioning calculation. Real-time correction of the positioning results is performed using algorithms like Kalman filtering.

By this modeling method, errors can be corrected in real time in environments with severe multipath effects, significantly enhancing positioning accuracy.

#### 2.1.4. Kalman filtering.

To achieve real-time correction of multipath errors, this study applies the Kalman filtering algorithm [31,32] to integrate correction signals with positioning data. Through a feedback mechanism, the filter parameters are adaptively adjusted to realize high-precision monitoring of slope deformation.

The fundamental principle of Kalman filtering is to obtain the optimal estimate based on the minimum mean square error criterion. By modeling the signal and noise in a state-space framework, the algorithm utilizes both the estimated state from the previous time step and the current observation to compute the optimal estimate at the current time step. The estimation process follows the formulation that minimizes the mean square error for the signal of interest. The Kalman filter operates iteratively through the following five equations:

Time Update Equations:


$${\hat{X}}_{k}^{-}={\hat{A}}_{k-1}+B{\hat{U}}_{k-1}$$ (3)

$$P_{k}^{-}=AP_{k-1}A^{T}+Q$$ (4)

Measurement Update Equations:


$$K_{k}=P_{k}^{-}H^{T}(HP_{k}^{-}H^{T}+R{)}^{-1}$$ (5)

$${\hat{X}}_{k}={\hat{X}}_{k}^{-}+K_{k}(Z_{k}-H{\hat{X}}_{k}^{-})$$ (6)

$$P_{k}=(I-K_{k}H)P_{k}^{-}$$ (7)

In the above equations: *A* is the *n* × *n* state transition matrix applied to $X_{k-1}$; *B* is the *n* × 1 control input matrix applied to the control vector $U_{k-1}$; *H* is the *m* × *n* observation model matrix that maps the true state space into the observed space; $P_{k}^{-}$ denotes the prior estimation error covariance matrix (*n* × *n*); $P_{k}$ denotes the posterior estimation error covariance matrix (*n* × *n*); *Q* is the process noise covariance matrix (*n* × *n*); *R* is the measurement noise covariance matrix (*m* × *m*); *I* is the *n* × *n* identity matrix; $K_{k}$ is the *n* × *m* Kalman gain or blending factor, which serves to minimize the posterior estimation error covariance.

The selection of the filtering model is a critical factor in determining whether the Kalman filter will remain stable or diverge. To ensure the rationality and accuracy of the results, the model should closely reflect the actual physical conditions. In deformation monitoring projects, the most commonly used Kalman filtering model is the position-velocity model. The state equation of the position-velocity filtering model is shown in Equation (8). In this model, the mean value of the displacement velocity of the monitoring point is assumed to remain constant. The position and displacement velocity of the monitoring point are treated as state variables, while the displacement acceleration is considered as dynamic noise.


$$\left[\begin{array}{c} {}*20cx_{k}\\ {\dot{x}}_{k} \end{array}\right]=\left[\begin{array}{cc} {}*20c1 & t\\ 0 & 1 \end{array}\right]\left[\begin{array}{c} {}*20cx\\ x_{k} \end{array}\right]+\left[\begin{array}{c} {}*20c\frac{1}{2}t^{2}\\ t \end{array}\right]\Omega_{k-1}$$ (8)

When the observation noise and process noise models are known, a dedicated accuracy assessment is not required. However, in practical applications, the adopted prior process noise covariance matrix *Q* and measurement noise covariance matrix *R* may not accurately reflect their true values—and in some cases, may deviate significantly. In such situations, it is more appropriate to regard *Q* and *R* as weight inverse matrices. Accordingly, the term $P_{k}$ in Equation (7) represents the weight inverse matrix of the system state vector. As a result, it becomes necessary to estimate the unit weight variance, which in turn allows for the computation of the covariance matrix of the estimated state parameters.

For the *k*-th epoch, the unit weight variance $\sigma^{2}$ can be estimated using the following formula:


$$\sigma_{k}^{2}=\frac{\xi_{k}^{T}P_{\xi_{k}}^{-1}\xi_{k}}{n_{k}}$$ (9)

$$P_{\xi_{k}}^{-1}=(HP_{k}^{-}H^{T}+R{)}^{-1}$$ (10)

$$\xi_{k}=Z_{k}-HX_{k}$$ (11)

In the equation, $n_{k}$ denotes the number of filtered observations at epoch *k*, and $P_{\xi_{k}}^{-1}$ represents the weight inverse matrix of the predicted residual vector $\xi_{k}$. At this point, the covariance matrix of the filtered state parameter vector can be computed accordingly.


$${\bar{P}}_{k}=\sigma^{2}*P_{k}$$ (12)

In deformation monitoring, $n_{k}$ is often relatively small, which may lead to instability in $\sigma_{k}^{2}$ estimated using Equation (9). Ideally, $\sigma^{2}$ should be estimated using the residual vector derived from the smoothed state parameter vectors across all epochs. However, smoothing calculations are computationally intensive and complex. To simplify the computation and mitigate the instability of $\sigma_{k}^{2}$, an approximate estimation $\sigma^{2}$ can be obtained by computing the weighted average of the unit weight variances $\sigma_{k}^{2}$ derived from each epoch using Equation (9). Let *N*_k_ denote the cumulative number of observations up to and including epoch *k*, and let $W_{k}$ denote the cumulative sum of the products $\xi_{k}^{T}P_{\xi_{k}}^{-1}\xi_{k}$ up to and including epoch *k*. Then, the following simplified formula can be derived:


$$N_{k}=N_{k-1}+n_{k}$$ (13)

$$W_{k}=W_{k-1}+\xi_{k}^{T}P_{\xi_{k}}^{-1}\xi_{k}$$ (14)

$${\bar{P}}_{k}^{2}=W_{k}N_{k}^{-1}$$ (15)

In summary, the general procedure for applying the Kalman filter to process monitoring data involves the following steps: First, collect monitoring data from a subset of monitoring targets to analyze and determine an appropriate model for the system state variables. Second, extract the process noise and measurement noise characteristics from the data to establish the Kalman filtering model and define the initial values of the system state vector. Finally, construct the system’s Kalman filtering framework and apply it to process the observational data.

### 2.2. Real-time error correction mechanism based on correction signals

#### 2.2.1. Concept and application of correction signals.

The BeiDou system enhances positioning accuracy by providing correction signals. A correction signal is a real-time broadcast signal that delivers precise satellite orbit and clock bias information to the receiver, thereby reducing positioning errors caused by the satellite system’s own inaccuracies. The real-time correction capability of correction signals plays a crucial role in dynamic monitoring, especially when correcting errors caused by multipath effects.

The PPP-B2b signal (B2b refers to the enhanced signal of the B2 frequency band) is a correction signal based on Precise Point Positioning (PPP) technology in the BeiDou satellite navigation system, capable of providing high-precision real-time orbit and clock bias data. By receiving the correction signal, the receiver can promptly correct errors, improving positioning accuracy.

#### 2.2.2. Implementation of the real-time error correction mechanism.

To achieve real-time error correction, correction signals are combined with real-time positioning data for error correction in dynamic environments. The specific steps are as follows:

Real-Time Data Acquisition: Real-time satellite positioning data is obtained using a BeiDou receiver and transmitted to a data center for processing.

Receiving Correction Signals: Based on satellite signal reception, the PPP-B2b correction signal is received in real time from the BeiDou system to obtain accurate satellite orbit and clock bias information.

Error Correction: Real-time error correction is performed using the correction signal. By incorporating correction signal adjustment terms into the positioning algorithm, errors caused by multipath effects and other interference factors are eliminated.

Accuracy Evaluation and Feedback: Real-time monitoring data is evaluated for accuracy, and the correction mechanism is adjusted based on error feedback to maintain the stability of monitoring precision.

Through this real-time error correction mechanism, the impact of multipath effects on positioning accuracy can be effectively addressed, ensuring high precision and reliability in slope deformation monitoring, especially in complex environments.

### 2.3. Implementation and optimization of multipath error processing technology

**Fig 2:**
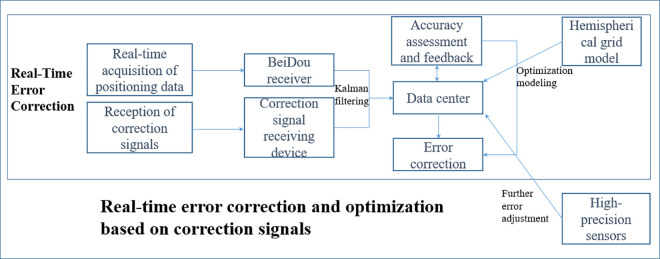
Real-time error correction and optimization based on correction signals.

To further improve accuracy, multipath error processing relies not only on a single correction signal but also on the integration of multiple data sources for comprehensive optimization. To enhance the effectiveness of error correction, this paper employs the multi-source data fusion technology shown in Fig 2, combining correction signals, real-time positioning data, and ground sensor data for error correction and precision optimization [33]. Specifically, through the Kalman filtering algorithm, the correction signal and positioning data are fused to correct multipath errors in real time. The filter parameters are adjusted through a feedback mechanism to achieve the optimal precision correction effect. Based on the aforementioned MHGM, multipath errors in different environments are modeled, and the correction model is continuously optimized to meet the various demands of slope deformation monitoring. During actual monitoring, the error correction mechanism is dynamically adjusted based on the dynamic characteristics of slope deformation to respond to changing environmental conditions.

## 3. Experimental design and data analysis

### 3.1. Experimental equipment and data acquisition

#### 3.1.1. Experimental equipment.

**Fig 3:**
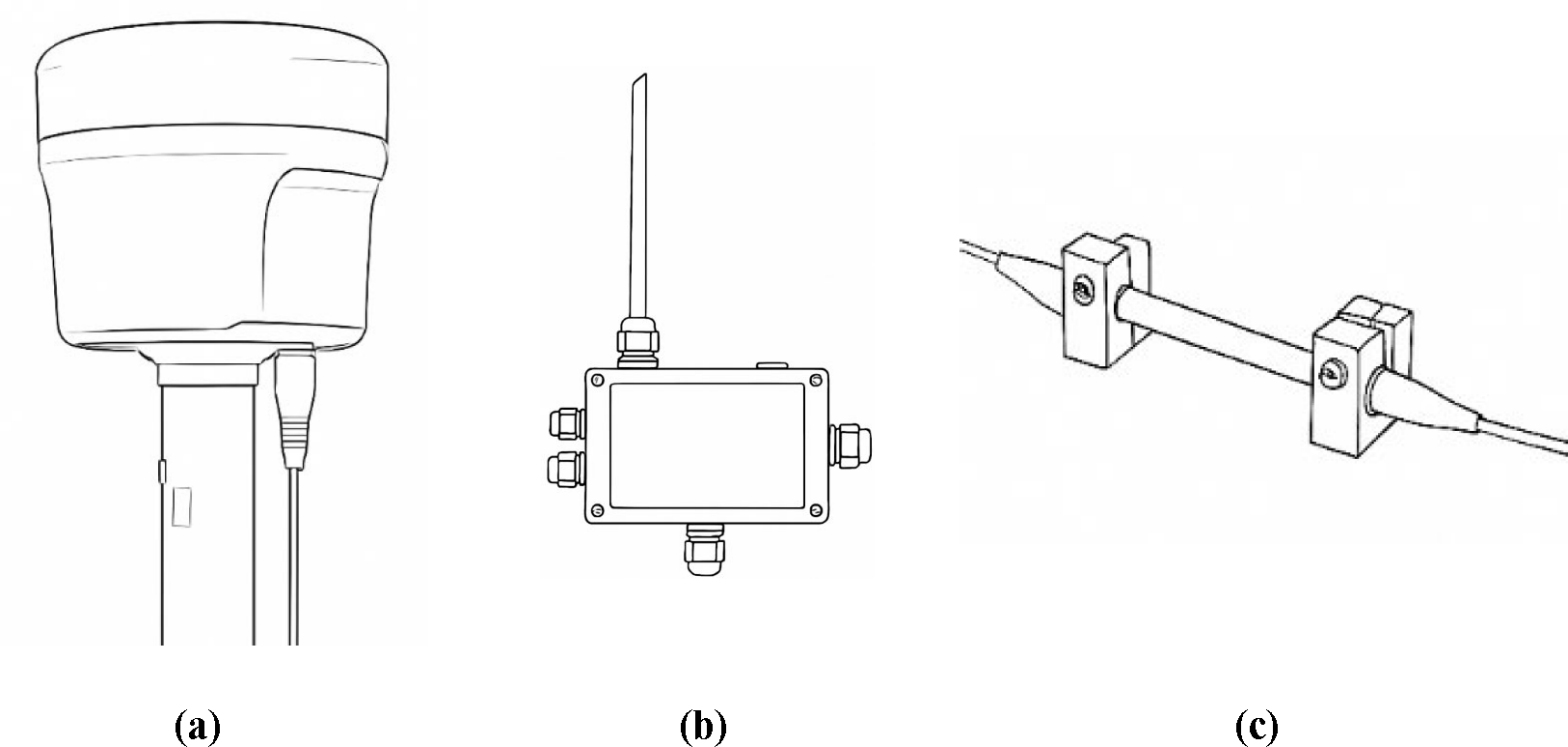
Main equipment used in the experiment: (a) Beidou receiver; (b) Correction signal reception device; (c) High-precision sensor.

The equipment used in the experiment includes the BeiDou receiver, correction signal reception device, and high-precision deformation sensors, as shown in Fig 3. The high-precision BeiDou receiver is used to collect satellite signal data in real time and perform positioning calculations. The correction signal receiver is used to receive the BeiDou PPP-B2b correction signal, providing precise satellite orbit and clock bias information. The deformation sensors are used to monitor slope displacement in real time and provide measured data for slope deformation. Additionally, computational and analysis equipment is used to process and analyze the real-time collected data, evaluating positioning accuracy and deformation monitoring precision.

As shown in Fig 3(c), high-precision deformation sensors typically achieve sub-millimeter accuracy. The installation process generally involves the following steps: Selection of Installation Location. An appropriate installation site is selected based on monitoring objectives and field conditions. Sensor Mounting. The sensor must be securely mounted at the designated measurement point to prevent errors caused by loosening or displacement. Special mounting holes are used to ensure that the sensor remains stable and unaffected by external disturbances during the monitoring period. Wiring and Connection. After installation, the sensor is connected to the data acquisition system via cables. Weather-resistant and interference-resistant cables are used, and proper cable fixing and protection are ensured to prevent physical damage or signal loss.

The calibration procedure includes the following steps: Zero Calibration. After installation, a zero-point calibration is performed by recording the sensor output under no deformation. This ensures the initial reading is zero, thereby eliminating bias due to temperature changes or other environmental factors. Load Calibration. A known load (e.g., standard force or standard deformation) is applied to the sensor. The output signal change is measured to calibrate the sensor’s sensitivity. By comparing the sensor’s response with the known standard, the output can be adjusted to ensure measurement accuracy. Temperature Compensation. Since the output of strain gauges is affected by temperature, compensation is required. Calibration is conducted under various temperature conditions, and real-time temperature monitoring is performed using dedicated temperature sensors to correct measurement errors caused by temperature fluctuations. Dynamic Calibration. For sensors used in long-term monitoring, periodic dynamic calibration is necessary to check for deviations caused by aging or environmental changes.

#### 3.1.2. Data acquisition process.

The data acquisition is divided into two parts: real-time positioning data collection and correction signal acquisition. For real-time positioning data collection, the BeiDou receiver continuously collects satellite positioning data to ensure a sufficient number of high-frequency data points, meeting the demand for real-time slope deformation monitoring. The correction signal is acquired concurrently with the positioning data: based on the BeiDou receiver’s collection, the PPP-B2b correction signal is received in real time to obtain precise satellite orbit and clock bias information. The collection of correction signals and positioning data is synchronized to ensure the effectiveness of real-time error correction. During the experiment, multiple monitoring points were established to enable simultaneous monitoring of different slope sections. Deformation data at each monitoring point were collected every 30 minutes. In addition, positioning data and correction signals were updated every 30 minutes in response to real-time changes in satellite signals. The total duration of data collection was 3,000 minutes.

### 3.2. Experimental environment and data analysis

#### 3.2.1. Experimental environment.

The experiment selected three typical highway slope environments for testing: Mountainous Environment: This environment features significant terrain fluctuations, with slopes composed mainly of rock or mixed soil. The terrain is complex, and satellite signals are easily affected by reflection and refraction from the mountain. Canyon Environment: The steep mountainsides and narrow spaces in the canyon lead to frequent reflections of satellite signals, which often cause strong multipath effects. Flat Area: This serves as a comparison test environment with simpler terrain and relatively mild multipath effects. For each type of environmental condition, 10 monitoring points were established on the highway slope for deformation monitoring.

#### 3.2.2. Data analysis methods.

To compare the effects of different technologies, several data analysis methods are employed:

Error Calculation and Accuracy Evaluation: The positioning errors using multipath error processing technology are compared with traditional methods (such as simple differential positioning) across different environments. The positioning accuracy (such as location accuracy and deformation monitoring accuracy) is calculated. The error evaluation formula is:


$$E_{rror}=\sqrt{{\left(X_{Act}-X_{Pre}\right)}^{2}+{\left(Y_{Act}-Y_{Pre}\right)}^{2}+{\left(Z_{Act}-Z_{Pre}\right)}^{2}}$$ (3)

Where, $X_{Act}$, $Y_{Act}$, $Z_{Act}$ and $X_{Pre}$, $Y_{Pre}$, $Z_{Pre}$ represent the actual measured and predicted deformation data, respectively.

Multipath Error Analysis: A comparison is made between the experimental group and the control group (the group not using error processing technology) regarding the error variations in different terrain environments, assessing the impact of multipath effects.

### 3.3. Experimental Procedure

(1) Preliminary Experiment: Slope deformation monitoring was conducted under three typical environmental conditions using conventional methods (e.g., differential positioning) without correction signals. Both positioning and deformation data were collected.
(2) Introduction of Correction Signals: Based on the conventional experiments, the PPP-B2b correction signal was introduced. Data were recollected with real-time error correction enabled.
(3) Data Fusion and Optimization: By integrating correction signals, real-time positioning data, and ground sensor measurements, multipath errors were corrected using the Kalman filtering algorithm.
(4) Data Comparison: A comparative analysis was performed across the three experimental conditions, focusing on positioning accuracy, deformation monitoring precision, and multipath error. Statistical analysis and accuracy assessments were conducted accordingly.

## 4. Data analysis and results discussion

### 4.1. Comparison of positioning accuracy

The positioning accuracy in three different environments was compared, with the experimental results shown in Table 1. For ease of comparison, the table lists the positioning accuracy using the traditional differential positioning method (without error correction technology) and the positioning accuracy after real-time error correction using the PPP-B2b correction signal.

**Table 1:** Comparison of positioning accuracy.

Environment Type	Traditional Method Positioning Error (cm)	Correction Signal Positioning Error (cm)	Positioning Accuracy Improvement (%)
Mountainous Area	6.31	2.12	66.40
Canyon Area	8.13	3.53	56.58
Flat Area	2.41	1.12	53.53

From Table 1, it can be observed that the positioning accuracy improved significantly in all environments after using the correction signal, with the most substantial reduction in positioning errors seen in the mountainous and canyon environments. This phenomenon indicates that the real-time error correction technology based on the BeiDou system is particularly effective in complex environments. In the mountainous and canyon environments, due to the reflection and refraction phenomena from the mountains or canyon walls, multipath effects on satellite signals are severe, leading to large positioning errors using traditional methods. After introducing the correction signal, real-time correction of these multipath errors significantly improved the accuracy.

### 4.2. Comparison of deformation monitoring accuracy

Next, the performance of different methods in deformation monitoring accuracy was compared, with the experimental results shown in Table 2. This table lists the deformation monitoring errors using traditional methods and those corrected by the correction signal.

**Table 2:** Comparison of deformation monitoring accuracy.

Environment Type	Traditional Method Deformation Error (cm)	Correction Signal Deformation Error (cm)	Deformation Accuracy Improvement (%)
Mountainous Area	5.79	2.31	60.10
Canyon Area	7.48	3.12	58.29
Flat Area	3.22	1.59	50.62

The data in Table 2 indicates that the correction signal significantly improved the accuracy of slope deformation monitoring, particularly in mountainous and canyon environments where deformation monitoring errors were notably reduced. Specifically, in the mountainous area, the error of the traditional method was 5.79 mm, which decreased to 2.31 mm after introducing the PPP-B2b correction signal, improving the accuracy by 60.10%. In the canyon environment, the error of the traditional method was 7.48 mm, which decreased to 3.12 mm after the correction signal, resulting in a 58.29% improvement in accuracy. In the flat area, although the improvement from the correction signal was relatively small, a noticeable accuracy enhancement was still observed, with the error reducing from 3.22 mm to 1.59 mm, a 50.62% improvement. These results fully validate the significant role of real-time error correction technology based on the BeiDou system in dynamic monitoring, especially in complex terrains, where the improvement in deformation monitoring accuracy is most pronounced.

### 4.3. Impact of multipath effects on positioning and deformation monitoring

**Fig 4:**
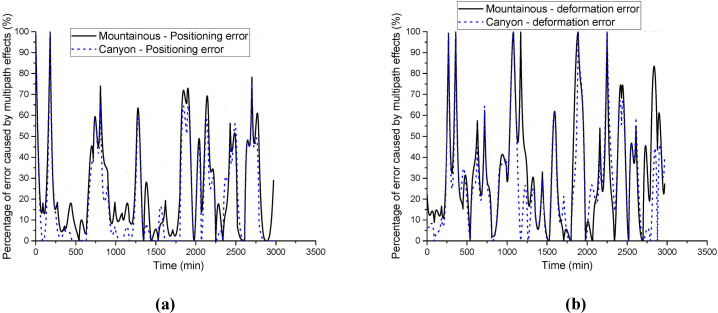
Impact ratio of multipath effects on positioning and deformation monitoring accuracy: (a) Positioning accuracy impact ratio; (b) Deformation accuracy impact ratio.

To further analyze the impact of multipath effects on the experimental results, the influence of multipath errors on positioning accuracy and deformation monitoring accuracy in different environments was calculated. The results are shown in Fig 4.

From Fig 4, it is clear that the impact of multipath effects on positioning and deformation monitoring accuracy is most prominent in mountainous and canyon environments. Combining the data from Table 1 and Table 2, it can be observed that in the mountainous environment, the positioning errors of traditional methods are primarily caused by multipath effects. The introduction of the correction signal effectively reduces the impact of multipath errors on positioning accuracy. In the canyon environment, multipath effects not only increase positioning errors but also contribute to increased deformation monitoring errors. However, with the real-time error correction mechanism, both positioning and deformation monitoring accuracy were effectively enhanced.

### 4.4. Analysis of real-time error correction effect

**Fig 5:**
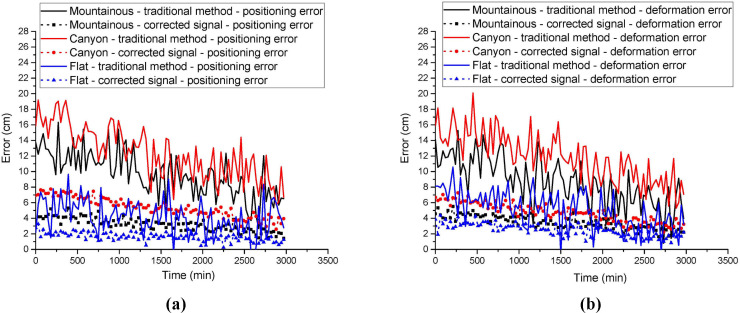
Variation of positioning and deformation accuracy over time: (a) Positioning accuracy; (b) Deformation accuracy.

To further verify the effectiveness of the real-time error correction mechanism, real-time positioning and deformation monitoring accuracy were evaluated under different experimental conditions. In the experiment, data were collected every 30 minutes at monitoring points, and positioning and deformation data were recorded. These data were compared with actual deformation, and positioning accuracy and deformation accuracy were calculated. The experimental results are shown in Fig 5.

From Fig 5, it can be seen that in the traditional method, due to the impact of multipath effects, both positioning accuracy and deformation accuracy exhibit significant fluctuations. After the correction signal was introduced, both positioning and deformation accuracy stabilized, and the fluctuation of errors significantly reduced. This indicates that the real-time error correction mechanism plays an important role in eliminating the impact of multipath effects and other interference factors, ensuring the high accuracy and reliability of monitoring data.

### 4.5. Analysis of the effectiveness of data fusion optimization

To further improve monitoring accuracy, a data fusion optimization experiment was conducted. By combining the correction signal, real-time positioning data, and ground sensor data, multipath errors were corrected using the Kalman filtering algorithm. The maximum correlation coefficients and root mean square errors (RMSE) of Kalman filtering in the three environments are summarized in Table 3. Mountainous Environment:Positioning: Maximum correlation coefficient = 0.96, RMSE = 1.7 cm. Deformation: Maximum correlation coefficient = 0.94, RMSE = 1.7 cm. This indicates that, in the mountainous environment, the Kalman filtering algorithm effectively corrects multipath errors, improving both positioning and deformation accuracy. However, despite these improvements, positioning and deformation accuracy are still affected by the complex terrain, leading to residual errors. Canyon Environment: Positioning: Maximum correlation coefficient = 0.94, RMSE = 2.5 cm. Deformation: Maximum correlation coefficient = 0.92, RMSE = 2.6 cm. In the canyon environment, the Kalman filtering algorithm performs less effectively compared to the mountainous environment. The maximum correlation coefficient is lower, likely due to the multipath and shielding effects inherent in the canyon environment. Flat Area: Positioning: Maximum correlation coefficient = 0.99, RMSE = 1.1 cm. Deformation: Maximum correlation coefficient = 0.98, RMSE = 1.6 cm. In the flat area, the Kalman filtering algorithm performs optimally, with the highest maximum correlation coefficient and the smallest RMSE. This suggests that the signal stability in flat areas is higher, allowing the Kalman filtering algorithm to effectively correct multipath errors, thereby enhancing positioning and deformation accuracy.

**Table 3:** Kalman filtering related statistical results.

Environment Type	Positioning – Maximum Correlation Coefficient	Deformation – Maximum Correlation Coefficient	Positioning – Root Mean Square Error (cm)	Deformation – Root Mean Square Error (cm)
Mountainous Area	0.96	0.94	1.7	1.7
Canyon Area	0.94	0.92	2.5	2.6
Flat Area	0.99	0.98	1.1	1.6

Mountainous Environment: The mountainous environment is characterized by complex terrain, where signal propagation is easily affected by obstruction and reflection from the mountain slopes. In this environment, multipath effects are more significant, leading to increased errors in positioning and deformation monitoring. Although Kalman filtering can correct these errors to some extent, the accuracy of positioning and deformation did not reach ideal levels due to the unique characteristics of the terrain. Notably, in deformation monitoring, the errors caused by complex terrain variations are more pronounced. Although the maximum correlation coefficient in this area is higher, the root mean square error (RMSE) remains significantly higher compared to the flat region.

Canyon Environment: The distinctive feature of the canyon environment lies in the multipath propagation of signals. The narrow space within the canyon and the reflective surfaces of the canyon walls cause positioning signals to become unstable, with reflected and refracted signals potentially contributing to multipath errors. This leads to a reduced effectiveness of the Kalman filter in this environment compared to the mountainous and flat regions, especially in deformation monitoring, where errors increase. Moreover, the shielding effects and signal attenuation in the canyon environment make real-time positioning more challenging, resulting in lower positioning and deformation accuracy in this environment.

Flat Area: Signal propagation in flat areas is more stable and less affected by obstruction and reflection, which allows the Kalman filter to effectively correct errors. Compared to the mountainous and canyon environments, the flat area experiences less signal interference, significantly improving positioning and deformation monitoring accuracy. The maximum correlation coefficient and RMSE in the flat area exhibit optimal results, proving that this environment is ideal for conducting experiments aimed at improving accuracy.

The positioning measurement errors and deformation measurement errors using traditional differential positioning methods (without error correction techniques) and those optimized by data fusion are shown in Table 4. From Table 4, it is evident that the accuracy is significantly improved after data fusion optimization, especially in complex environments, where the accuracy improvement exceeds 60%. Through real-time optimization with algorithms such as Kalman filtering and the fusion of multi-source data, not only was the positioning accuracy enhanced, but the accuracy of deformation monitoring was also improved. This further demonstrates the critical role of data fusion optimization in slope deformation monitoring.

**Table 4:** Accuracy improvement after data fusion optimization.

Environment Type	Traditional Method Positioning Error (cm)	Optimized Positioning Error (cm)	Positioning Accuracy Improvement (%)	Traditional Method Deformation Error (cm)	Optimized Deformation Error (cm)	Deformation Accuracy Improvement (%)
Mountainous Area	6.31	1.82	71.16	5.79	1.92	66.84
Canyon Area	8.13	2.49	69.37	7.48	2.81	62.43
Flat Area	2.41	1.02	57.68	3.22	1.49	53.73

### 4.6. Summary and discussion

From the above analysis, it can be seen that the multipath error processing technology based on the BeiDou system proposed in this paper effectively improved the accuracy of slope deformation monitoring in complex environments. Particularly in mountainous, canyon, and other complex terrains, the impact of multipath effects on monitoring results is significant. However, by introducing the PPP-B2b correction signal for real-time error correction, the positioning and deformation monitoring accuracy were significantly improved. Furthermore, data fusion optimization further enhanced the accuracy and stability of the monitoring system, providing strong technical support for highway slope deformation monitoring.

Based on the experimental results, the proposed technology demonstrated high effectiveness and adaptability across different environments. Especially in terrains with significant multipath effects, the monitoring accuracy was substantially improved, meeting the demands of modern highway slope deformation monitoring and providing valuable references for research and applications in related fields.

## 5. Engineering application

### 5.1. Project overview

**Fig 6:**
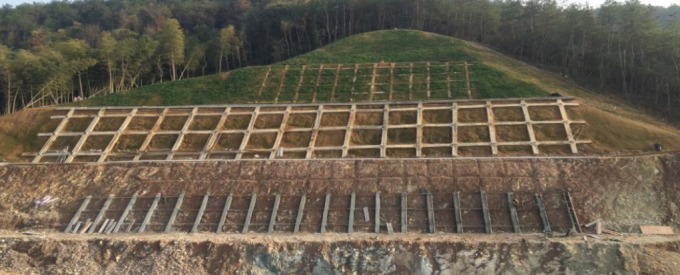
Application of engineering practices.

There are approximately 100 high slopes along the Hangzhou-Shaoxing-Taizhou Expressway. One of these slopes, as shown in Fig 6, was selected to monitor deformation using the method proposed in this paper. This high slope has a road length of about 160 meters and a slope height of approximately 41.21 meters, classified as a Grade V high slope, with 2-meter wide platforms set every 8 meters of height. The route in this cut-and-fill area is located in the low mountain and hilly slope zone at the edge of the Shengzhou Basin, with an overall mountain orientation from north to south. The route runs perpendicular to the mountain trend, passing through a long, narrow mountain. The mountain top is circular with a gentle slope, and the surface vegetation is well-developed. At the foot of the slope, there are two small reservoirs. The surface is sparsely distributed, mainly consisting of silty soil that is grayish-yellow in color with a thickness of 3–4 meters, exposing the surface of highly weathered ignimbrite rock.

Due to the influence of regional faults, significant differences in weathering are observed on both sides of the road centerline. On the north side of the centerline, the thickness of the highly weathered rock is thin, while the medium-to-lower weathered bedrock is relatively hard. The medium-to-upper weathered rock is relatively fragmented, mainly in the form of debris and short columns, with poor integrity. The deeply weathered rock is relatively intact, with cores mostly in short or columnar shapes. On the south side of the centerline, the rock is relatively softer, with deep weathering. The highly weathered layer is grayish-yellow, with a borehole exposure depth of 2.5 meters. The joints are extremely dense, and the highly weathered rock is mostly gravel-like, with local sandy soil. The fracture surfaces are mostly filled with clay. Beneath this layer, there is medium weathered ignimbrite, purple-gray in color, relatively hard, with clear ignimbrite structure and dense joints. The core is fragmented and in debris form, with kaolinite filling the fracture surfaces, exhibiting poor cementation.

The cut-and-fill area is located at the edge of the Shengzhou Basin’s low mountain and hilly area, where intense regional faulting has caused severe weathering of the rock and fragmentation of the rock mass, resulting in pronounced differential weathering. The vertical joints in this area are well-developed, with a strike of 5º∠78º, long and straight, closed and extending, densely developed, and the rock mass is fractured. The hydrogeological conditions in this area are relatively simple, with groundwater mainly coming from the weathered rock fractures and structural fractures in the rock mass. The water volume is quite limited, and special attention must be paid to the potential for sliding along the bedding plane when excavating the slope. Both the highly weathered ignimbrite and the medium weathered ignimbrite are soft rocks with poor engineering geological properties. They are weakly permeable, highly hydrophilic, and, when wet, the rock mass strength decreases, making it susceptible to deformation, particularly under rainy weather conditions.

The anchor bolt frame beam protection system allows for simultaneous excavation and protection, offering advantages such as saving engineering materials, reducing project costs, improving slope greening, and facilitating dynamic design. Therefore, this type of support system is chosen for the slope. Due to its complex terrain and diverse environmental factors, traditional deformation monitoring methods (such as optical measurements and manual leveling) are often influenced by weather, topography, and equipment limitations, leading to monitoring accuracy that fails to meet the requirements for real-time data and high precision. To enhance the accuracy and reliability of deformation monitoring, high-precision positioning and real-time error correction technologies based on the BeiDou system are applied.

### 5.2. Application of multipath error processing technology based on the BeiDou system

**Fig 7:**
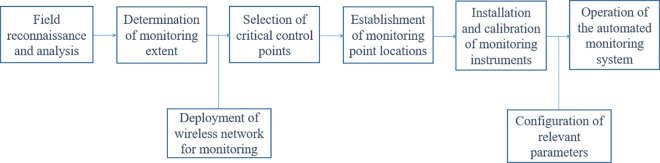
Monitoring system setup process.

A deformation monitoring system for the highway slope was established using the system setup process shown in Fig 7. Multiple BeiDou satellite receivers, combined with PPP-B2b corrected signals and a real-time error correction mechanism, were used to dynamically correct the impacts of multipath effects through multipath error processing technology. The monitoring data and corrected signals are transmitted to the data center via a communication network, where the deformation monitoring accuracy is further optimized through the Kalman filtering algorithm. Additionally, by incorporating the Multi-Hemispherical Grid Model (MHGM) and real-time data calibration, the system’s adaptability in dynamic environments is further enhanced.

**Fig 8:**
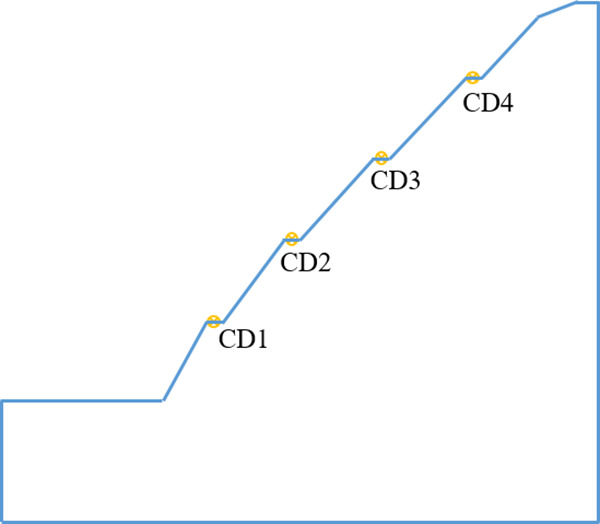
Layout of monitoring points.

Four monitoring points, as shown in Fig 8, are set up on the slope, with one monitoring point placed on each platform level to monitor horizontal and vertical displacements of the ground surface during construction. After the slope excavation has stabilized, a monitoring plan is implemented. A three-month field monitoring analysis is conducted, during which the effects of the entire plum rain season on the slope are observed.

### 5.3. Analysis of MONITORING Results

**Fig 9:**
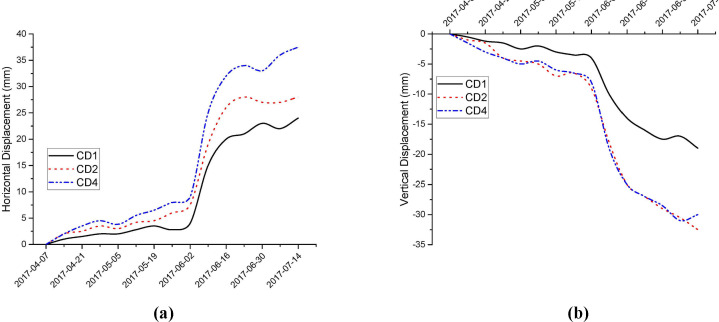
Main monitoring results: (a) Horizontal displacement; (b) Vertical displacement.

The monitoring point CD3 was damaged, and the collected data was incomplete. Therefore, the analysis was conducted only on the data from CD1, CD2, and CD4. The horizontal displacements and vertical displacements obtained from the monitoring are shown in Fig 9(a) and Fig 9(b), respectively.

The displacement curve corresponding to point CD1 indicates that the displacement at this point is small, with a maximum displacement reaching 25 mm. During the monitoring process, the changes in horizontal and vertical surface displacements are generally consistent. A comprehensive analysis shows that the displacement changes at this point are caused by both horizontal and vertical surface displacements. Due to the relatively hard rock quality, there is little change in displacement when there is no rainfall, suggesting that the slope on this side is relatively stable. However, under the influence of rainfall, a significant increase in both horizontal and vertical surface displacements was observed, particularly during the early part of the rainy season, with displacement reaching up to 13 mm, indicating a clear impact of rainfall. As the rainy season ended, the displacement changes at this point began to stabilize, with a trend of contraction in displacement changes.

Point CD2 is located on the left side of the platform in the soil section of the slope, which is more prone to deformation. From the displacement curve, it can be seen that the displacement at this point is significant, with a maximum displacement reaching 30 mm. During the monitoring process, the changes in both horizontal and vertical surface displacements are generally consistent. However, field observations revealed that this point exhibited surrounding settlement, with local cracks, where the crack width is less than 1 cm. The settlement phenomenon was more pronounced than the cracks. A comprehensive analysis shows that the displacement changes at this point are mainly caused by vertical displacement.

In the early part of the rainy season, the surface horizontal and vertical displacements at this point increased significantly, with displacements reaching up to 18 mm in the early rainy season. This is likely due to the nature of the soil material, where the appearance of cracks accelerates the infiltration of rainwater. After immediate reporting, the construction unit filled in the cracks in all areas and re-implemented drainage measures. As the rainy season ended, the displacement at this point gradually stabilized, though the deformation remained greater than that observed under conditions unaffected by rainfall. After the repairs were completed, and with the end of the rainy season, the displacement changes stabilized.

The displacement curve corresponding to point CD4 shows that the displacement at this point is significant, with a maximum displacement reaching 39 mm. During the monitoring process, the horizontal surface displacement was greater than the vertical surface displacement. A comprehensive analysis indicates that the displacement changes at this point are primarily caused by horizontal surface displacement. Field observations also revealed multiple cracks around the platforms at this point, with crack widths of up to 2 cm.

In the early part of the rainy season, both horizontal and vertical surface displacements at this point increased significantly. This increase may be attributed to intense rainfall infiltrating through the cracks, leading to a sliding tendency. The cracks were found to be as wide as 5 cm in some places. Prolonged heavy rainfall caused the drainage measures in the area to fail, resulting in the formation of deep gullies nearly 1 meter in depth. The construction unit subsequently filled the cracks and re-implemented drainage measures. As the rainy season ended, the displacement changes at this point gradually stabilized.

### 5.4. Application analysis

The application of this technology in the mountainous highway slope deformation monitoring project not only improved monitoring accuracy but also enhanced the system’s reliability and real-time response capabilities. By introducing correction signals in real time, the system was able to effectively handle complex multi-path effects, ensuring that deformation monitoring data remained highly accurate even in dynamically changing environments. Additionally, the system’s automation and real-time data processing capabilities enabled engineering personnel to obtain timely dynamic data on slope deformation, providing strong support for the highway’s safety management and early warning systems.

During the implementation of the project, the real-time and accuracy of monitoring data were significantly improved, ensuring that slope deformation monitoring met the needs of both engineering construction and safety management. These results provide valuable experience for similar engineering applications and demonstrate the great potential of the BeiDou system-based multi-path error processing technology in highway slope monitoring.

## 6. Conclusion

This study focused on improving the accuracy of highway slope deformation monitoring based on the BeiDou system. An innovative method for handling multi-path effects in the BeiDou system was proposed, and through real-time error correction mechanisms and the introduction of correction signals, the influence of multi-path effects on positioning and deformation monitoring accuracy was significantly reduced. The main research conclusions are as follows:

(1) This study innovatively proposed a real-time error correction mechanism based on correction signals, using the BeiDou PPP-B2b correction signal to correct multi-path errors in real time. Experimental results showed that, after introducing correction signals, not only were the positioning errors caused by multi-path effects effectively eliminated, but the accuracy of slope deformation monitoring was also improved. In mountainous and canyon environments, the positioning errors were reduced by 66.40% and 56.58%, respectively, and the deformation errors were reduced by 60.10% and 58.29%, respectively.
(2) By combining correction signals, real-time positioning data, and ground sensor data, the Kalman filtering algorithm was used to optimize the correction of multi-path errors. Through multi-source data fusion, experimental results showed that the optimized positioning accuracy improved by 71.16%, and deformation monitoring accuracy improved by 66.84%. This optimization approach demonstrated stronger adaptability and accuracy improvement, particularly in regions with significant multi-path effects, such as mountainous and canyon areas.
(3) The experiment was conducted in three typical environments (mountainous, canyon, and flat areas). The experimental results showed that the real-time error correction technology based on the BeiDou system effectively improved positioning and deformation monitoring accuracy in various environments, especially in complex terrains, where the introduction of correction signals significantly reduced the negative impact of multi-path effects on monitoring accuracy. Compared to traditional methods, the real-time correction mechanism with correction signals showed significant improvements in both positioning and deformation monitoring accuracy.
